# Long-term organogenic callus cultivation of *Ranunculus illyricus* L.: a blueprint for sustainable *ex situ* conservation of the species in urban greenery

**DOI:** 10.1186/s12870-024-04901-3

**Published:** 2024-03-25

**Authors:** Dawid Kocot, Barbara Nowak, Ewa Sitek

**Affiliations:** 1 Department of Ornamental Plants and Garden Art, Faculty of Biotechnology and Horticulture, University of Agriculture in Krakow, 29 Listopada 54, Krakow, 31-425 Poland; 2 Department of Botany, Physiology and Plant Protection, Faculty of Biotechnology and Horticulture, University of Agriculture in Krakow, 29 Listopada 54, Krakow, 31-425 Poland

**Keywords:** Buttercup, in vitro, Indirect organogenesis, Micropropagation, Picloram, Ranunculaceae, Vegetative explants

## Abstract

The growing trend of introducing wild plant species into urban environments necessitates the identification of novel species adapted to prevailing conditions. A promising reservoir of such species may be xerothermic communities where *Ranunculus illyricus* occurs. This study aimed to establish a micropropagation protocol for *R. illyricus* using indirect organogenesis. The protocol includes initiation of culture from various explants, callus proliferation, shoot regeneration, multiplication, and concurrent rooting. Callus appeared on most types of vegetative explants tested, but stolons were considered the best due to their good availability, high disinfection (85%), and robust callus production (maximum increase − 363.1%). The growth rate of the callus fresh matter (CFM) obtained from stolons was calculated. Greater CFM was obtained on the medium with the supplemented picloram 8.0 mg L^− 1^ with kinetin 5.0 mg L^− 1^ and in second part of experiment on medium with the addition of 2,4-D (2,4-dichlorophenoxyacetic acid) 2.0 mg L^− 1^ alone or picloram 6.0 mg L^− 1^ with kinetin 8.0 mg L^− 1^. Shoot organogenesis was observed on macronutrients B_5_ (Gamborg medium), micronutrients MS (Murashige and Skoog) medium with the addition of 2.0 mg L^− 1^ IBA (indole-3-butyric acid) and 4.0 mg L^− 1^ BAP (6-benzylaminopurine). To document the process of callus differentiation, microscopic preparations were prepared. Subsequently, the regenerated plants underwent acclimatisation and their growth in an *ex situ* collection was monitored over three growing seasons. In particular, in vitro-origin plants exhibited developmental patterns similar to those of their seed-origin counterparts. The incorporation of *R. illyricus* into urban landscapes not only increases aesthetic appeal, but also ensures the preservation of valuable genetic resources for this rare species, potentially contributing to effective *ex situ* conservation in the future. This marks the first scientific report on in vitro cultures of *R. illyricus*.

## Background

In the time of global climate change and high anthropopressure, plants that are evolutionarily adapted to survive in an environment with a high level of abiotic stress (extremophytes) may find more and more applications [1, 2]. Increasingly frequent extreme abiotic conditions in cities create conditions for the growth of species from thermophilic grass communities, i.e., extremely dry and photophilous. A rich source of valuable species with high decorative value that can be successfully cultivated on sites with poor, periodically dry soil and high temperatures are xerothermic grasslands.

Introducing xerothermic species in selected urban spaces can be an alternative to lawns or meadows that require a lot of care treatments [3]. Local wild species can be used in urban spaces as a complement to a selection of species in terms of colour or texture. Wildflowers in green spaces are not only visually attractive, but also create islands and corridors of biodiversity, both in rural [4] and urban [5] spaces.

New habitats in rapidly growing urban spaces (road verges, vacant lots, wastelands, parks, and gardens) not only increase biodiversity but also can preserve native plant species threatened with extinction. In developing urban spaces, the growth of ecological and cultural richness is conducive to the development of places of creative protection [6].

**Fig. 1:**
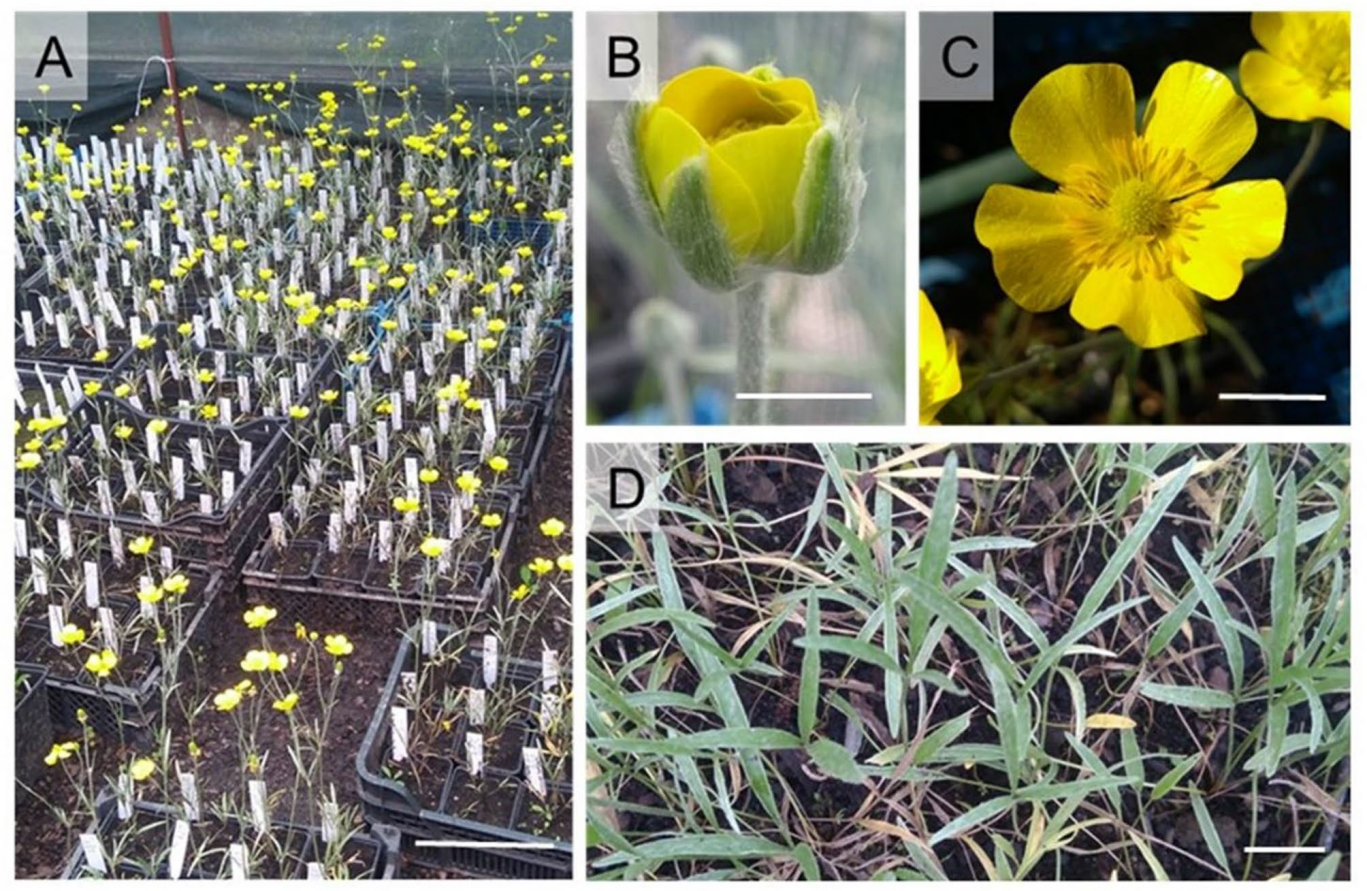
*Ranunculus illyricus* in the collection of the University of Agriculture in Kraków. (**A**) individuals at the beginning of the blooming period, bar = 15 cm; (**B**) initial flowering stage; (**C**) optimal flowering stage; (**D**) leaves, B-D) bar = 1 cm

One of the species representing floristically a very rich habitat – flowery xerothermic grasslands – is *Ranunculus illyricus* L. (Illyrian buttercup). Its flowers stand out with a very vivid yellow colour and can be used to create strong contrasts. The characteristic leaves covered with tomentose hair giving the original texture create a contrast to the green of other species (Fig. 1). Due to its features, this species can be recommended especially in the design of perennial meadows in the note of leading aspects, as a strong, periodic dominant [7].

*R. illyricus* is a geophyte with two modes of reproductions: generatively and vegetatively by stolons developing from the middle of tubers cluster formed at the base of shoot. The species is found in parts of Europe and Asia. In some European countries, especially where it occurs in dispersed populations, it is a rare and protected species [7]. In Poland, it had been considered an extinct species until 2001 when it was rediscovered. Currently, there are two populations of this species in Poland [8, 9].

Among numerous species of the genus *Ranunculus* (including nearly 600 species of perennials or annuals) [10, 11], relatively few have been propagated in vitro so far. The first works with *R. sceleratus* described the morphogenesis of floral buds in in vitro conditions followed by callus formation and organo- and embryogenesis [12–14]. This species, like many others in this genus, is rich in valuable secondary metabolites and more recent works focused on the application aspects of plants obtained through embryogenesis [15].

Many species of the genus *Ranunculus*, being endemic, are endangered because of their medicinal values and overexploitation. These species deserve to be propagated in vitro, given their biochemical composition and conservation measures. For these reasons, a method for propagating *R. walliachanus* in shoot cultures was developed [16, 17]. For *R. serbicus*, the method of suspension culture and regeneration by embryogenesis as a source to obtain secondary metabolites was described [18]. In turn, embryogenesis [19] and indirect organogenesis protocols [20] were developed for the endemic species *R. casusensis*.

To date, however, little effort has been made to enhance this genus for horticultural use through controlled hybridisation, selection, and multiplication of superior forms, except for *R. lyallii* propagated using shoot cultures [21]. Most of the attention in research work has been paid to *R. asiaticus*, a species of ornamental value, mainly as a cut flower, but also as a flowering potted plant [22, 23]. Given the high commercial importance of the species, attempts have been made to develop effective propagation systems in vitro [24, 25]. Callus cultures also enabled the induction of somatic embryos and the observation of their further development depending on the growth regulators applied [26, 27], shoot density and type of sugar [28] or agar [29] used.

In vitro techniques have great potential in breeding investigations [30] and are very useful when introducing new varieties or clones to the market. This method of cloning results in healthier and more uniform plants [31, 32].

The aim of our study was to develop a method of in vitro propagation of *R. illyricus* in conservation of species resources and as an alternative *ex situ* protection strategy. Obtained plants through in vitro cultivation can be used to evaluate potential of the species for enhancing biodiversity within urban landscapes. Additionally, this protocol has high potential for deliberate cross-breeding aimed at developing novel hybrid buttercup cultivars with an increased number of flowers per shoot, particularly catering to the cut flower market.

## Materials and methods

### Plant material

The plant material for culture initiation was collected from an *ex situ* population of Illyrian buttercup (*R. illyricus*) (Fig. 1) grown in a collection at the Faculty of Biotechnology and Horticulture of the University of Agriculture in Kraków, Poland. The plants were received from the Botanic Garden of the Jagiellonian University in Kraków and represented one of the Polish natural populations from Miernów (50°20′N 20°35′E, Świętokrzyskie Voivodeship, Poland) [7, 8].

### Initiation of culture

The material used to establish in vitro cultures comprised seeds, apical buds, flower receptacles and various vegetative explants: leaves, petioles, underground stolons, and underground tubers. Seeds sowing took place immediately after harvest on July 2, 2014. The sowing material was in the form of single-seeded achenes. Apical buds were taken from new plants growing froma cluster of vegetative tubers and approximately 2 cm in height in spring 2015. Tubers were collected in autumn 2014 after the end of the dormant period of the plants [33], whereas leaves, petioles, and stolon fragments were collected in spring 2015 and 2016 from young plants. The leaves with petioles had length of 5–6 cm, and the stolons of about 10 cm. The standard size of the single tuber used as explant was 4 mm. A flower receptacle was extracted from a bud 1–2 days before flowering in 2016.

#### Seeds

The sown achenes were disinfected for 30 s in 70% ethyl alcohol, 3.5 min in 0.1% mercuric chloride with a drop of Tween and then rinsed three times in sterile distilled water. The decontaminated achenes were placed on S-sowing medium (abbreviated as S medium), consisting of MS – Murashige and Skoog - mineral medium [34] with an addition of 10 g of sucrose, and 8.0 g L^− 1^ of agar (Agar-Agar, BTL- Łódź, Poland), pH 5.8; 5 pieces in each Petri dish with a diameter of 8 cm. A total of 65 achenes were sown: 35 on an S medium with an addition of GA_3_ (Gibberellic acid, Sigma) at a concentration of 1.0 mg L^− 1^; 40 on medium without an addition of GA_3_, but half of them germinated at 10 °C. Two factors have been tested to break dormancy: the use of gibberellin and the low germination temperature (Table 1). Petri dishes with disinfected achenes were kept under a 16/8 h day/night photoperiod and a photon flux density of 70 µmol m^− 2^ s^− 1^, cool white light (LUMILUX, L 36 W/840) Osram, Germany,

**Table 1:** Germination conditions of *R. illyricus* seeds on S medium

Combination of factors	Seed number	GA_3_ supplementation	Temperature of germination
Control	20	-	24 ± 2 °C
1.0 mg L^− 1^GA_3_	25	+	24 ± 2 °C
Low temperature of germination	20	-	10 °C

a temperature of 24 ± 2 °C in a growing room or a temperature of 10 °C in a growing chamber.

#### Fragments of plants

The apical buds and vegetative parts of plant mentioned in topic 2.2, after rinsing in water with a detergent, were disinfected in ethyl alcohol for 1 min and then for 5 min in mercuric chloride with Tween, then rinsed three times in sterile water and cut into 10 mm long explants. The outer leaves were removed from the disinfected shoot tips to expose the apical buds. The number of explant repetitions used for callus initiation depended on the availability of given organs.

The explants were placed individually in tubes (apical buds) or 100 ml Erlenmeyer flasks on callus growing medium (abbreviated as K basal medium), containing Gamborg macronutrients B_5_ [35], micronutrients MS with 2.0 mg L^− 1^ of glicyne, 1.0 mg L^− 1^ of thiamine, 0.5 mg L^− 1^ of pirydoxyne, 0.5 mg L^− 1^ of nicotinic acid, 100 mg L^− 1^ of myo-inositol, 30 g L^− 1^ of sucrose and 8.0 g L^− 1^ of agar. The K basal medium was supplemented with different combinations of plant growth regulators - PGR (Table 2), pH was adjusted to 5.8 before autoclaving at 121 °C for 20 min. Tubes with shoot tips were kept under a 16/8 h day/night photoperiod, while Erlenmeyer flasks with the other types of explants were kept in darkness in a growing room at a temperature of 24 ± 2 °C. The disinfection and callus growth results were evaluated after six weeks, the callus was subcultured and after obtaining a sufficient volume it was used to set up the experiment.

**Table 2:** Types of initial explants and plant growth regulators used to initiate *R. illyricus* cultures in vitro

Type of explant	Auxins (mg L^− 1^)	Cytokinins (mg L^− 1^)	Number of explants
Apical buds	NAA^*^ 3.2	Kinetin 5.0	7
	NAA 2.5	BAP^**^ 2.5	4
Leaf	Picloram 8.0	Kinetin 3.0	6
	Picloram 8.0	Kinetin 5.0	22
	Picloram 8.0	Kinetin 8.0	20
	NAA 5.0	BAP 5.0	7
	NAA 2.5	BAP 2.5	3
Petioles	Picloram 8.0	Kinetin 5.0	2
	Picloram 8.0	Kinetin 8.0	3
	NAA 5.0	BAP 5.0	6
	NAA 2.5	BAP 2.5	3
Stolons	Picloram 8.0	-	10
	Picloram 8.0	Kinetin 3.0	13
	Picloram 8.0	Kinetin 5.0	14
	Picloram 8.0	Kinetin 8.0	14
	2,4-D^***^ 4.0	Kinetin 8.0	11
	NAA 5.0	BAP 5.0	5
	NAA 2.5	BAP 2.5	5
Flower receptacle	Picloram 8.0	Kinetin 5.0	3
	Picloram 8.0	Kinetin 8.0	6
Tubers	2,4-D 4.0	Kinetin 8.0	34

^*^ NAA − 1-Naphthaleneacetic acid (Sigma), ^**^BAP − 6-Benzylaminopurine (Duchefa), ^***^2,4 –D − 2,4-Dichlorophenoxyacetic acid (Sigma)

### Callus cultures and indirect organogenesis

#### Experiment 1 - callus growth

The callus obtained from the stolon fragments cultured on medium supplemented with 8.0 mg L^− 1^ of picloram and 5.0 L^− 1^ of kinetin was used in the callus propagation experiment. Its growth was compared on K basal medium with the addition of 8 mg L^− 1^ picloram (Sigma) and 5 mg L^− 1^ or 8 mg dm^− 3^ kinetin (Sigma). The callus was subcultured every 5–6 weeks; at the beginning of the subculture the fresh matter of the callus and at the end of the subculture the fresh and dry matter were estimated. The dry matter of the callus was assessed according to the Rennert [36] protocol and presented as a percentage of the fresh matter. The growth rate of the callus fresh matter (CFM) was calculated according to the following formula:

*CFM% = (FMf – FMi)/FMi × 100%*.

where.

CFM% - callus fresh matter gain in %,

FMi - initial fresh matter of callus (mg),

FMf - final fresh matter of callus (mg).

The experiment involved ten 100 ml Erlenmeyer flasks (repetitions), each with five callus pieces of the total initial weight of 640–830 mg per flask. The flasks were kept in a phytotron in continuous darkness at a temperature of 24 ± 2 °C.

#### Experiment 2 - callus growth and organogenesis

The adventitious buds were induced in the callus. Five pieces of callus with a diameter of approximately 5 mm were placed on K basal medium supplemented with different combinations of plant growth regulators (Table 3). The experiment was carried out for three consecutive subcultures with six flasks per combination in the first subculture, 6–16 flasks per combination in the second subculture, and 5–20 flasks per combination in the third subculture. The number of flasks (repeats) for the combination in the second and third subsequent passages depended on the availability of callus resulting from its growth rate. The callus was subcultured every 5–6 weeks and weighted at the beginning and end of subculture. The CFM growth rate was evaluated for each flask. The flasks were kept in a phytotron in continuous darkness.

**Table 3:** Combinations of plant growth regulators in media for *R. illyricus* callus growth and differentiation

Growth regulators (mg L^− 1^)	
Auxins	Cytokinins
-	-
Picloram 4.0	Kinetin 8.0
Picloram 6.0	Kinetin 8.0
IBA* 2.0	BAP 4.0
IBA 1.0	BAP 4.0
2,4-D 2.0	-

^*^ IBA - Indole-3-butyric acid (Sigma)

### Multiplication and rooting of the shoots

The adventitious buds regenerated were excised and moved onto K basal medium supplemented with 1.0 mg L^− 1^ IBA and 2.0 mg L^− 1^ BAP. The Erlenmeyer flasks with five explants were kept for a 16/8 h day/night photoperiod, a photon flux density of 70 µmol m^− 2^ s^− 1^ and a temperature of 24 ± 2 °C. After six weeks of cultivation, the multiplication of shoots (number of shoots) and the efficiency of rooting were evaluated for 4 randomly selected flasks in 4 consecutive passages.

### Hardening and acclimatisation of regenerates

Seventy-four well-developed and rooted shoots were isolated from the medium with 1.0 mg L^− 1^ IBA and 2.0 mg L^− 1^ BAP (mentioned in 2.4. chapter). All were washed with tap water to remove any traces of agar and planted in multipots containing a 1:2 mixture of perlite and peat substrate. Hardened plantlets were grown for 8 weeks in Sanyo vegetative chambers (San-Yoonoda, Japan), under a 16/8 h day/night photoperiod and a photon flux density of 70 µmolm^− 2^ s^− 1^, a temperature of 24 ± 2 °C, with gradually reduced humidity from 70%. After 2 months, the acclimatised plantlets were finally transferred to separate pots with a diameter of 7 cm filled with a 1:2 mixture of perlite and commercially available potting soil AURA® from Agaris Poland under open field conditions. Since the April of 2020 they were cultivated in the collection at the Faculty of Biotechnology and Horticulture of the University of Agriculture in Kraków which is located within the urban area in a sunny place. According to recorded thermal conditions the average day temperature of April was 10 °C (min. 0 °C max. 30 °C). In September 2020, the dormant underground parts of the plants (tubers) were removed from the substrate, and the formed tubers were counted before they were replanted. That observation was repeated in the following season (2021).

### Histological studies

To trace and document the differentiation processes that take place in the callus tissue, microscopic preparations were made using objects randomly collected from various stages of experiments (callus with differentiating adventitious buds or developed structures). The objects were fixed in glutaraldehyde [37]. After dehydration of the material in an increasing percentage concentration of ethyl alcohol (30, 50, 75, 96 and 100%) and acetone, the tissue was saturated with a mixture of acetone and Epon, and then embedded in resin (Epoxy embedding, Sigma) [38]. The blocks were cut with glass knives using a Tesla 490 A ultramicrotome in sections with a thickness of approximately 1 μm. The preparations were stained with a 1% methylene blue aqueous solution.

### Ploidy assessment

Flow cytometric- based plant ploidy analysis was performed in the Cytogenetics Laboratory of the Sugar Beet Breeding Station in Kutno (Poland). The ploidy level was determined for samples of young leaves collected separately from each plant and prepared according to the Galbraith method [39] modified by Thiem and Śliwińska [40]. The chopped plant material in 2 mL of lysis buffer with an addition of DAPI fluorochrome dye was filtered and analysed using a PartecCyFlow Ploidy Analyser (Sysmex). A random selection of ten in vitro regenerated plants was evaluated, the seed-origin plant served as a reference.

### Photographic documentation

Anatomical preparations were photographed using a Nicon camera, while macrophotographs from the Image Pro Plus 4 Program were taken with a Canon camera or with a Zeiss Discovery stereoscopic magnifier using the Axio IMager 4.8 program (Carl-Zeiss Imaging Systems).

### Statistical analysis

The number of repetitions (flasks) was specified for each stage of the in vitro experiments in the methodology. The results were evaluated using the one-way ANOVA module in STATISTICA ver. 13 (StatSoft Inc, Tulsa, OK, USA). A post-hoc mean separation test was performed using the Tukey test at *P* ≤ 0.05.

## Results

### Initiation of culture

#### Seeds

None of the seeds sown on the medium germinated, regardless of the dormancy-breaking factor used.

#### Fragments of plants

All apical buds taken in the first passage seemed to be decontaminated,. however further cultivation in the second and third passages revealed developing infections that gradually eliminated the initially decontaminated buds. The effectiveness of the disinfection of the remaining plant fragments was in the range of 70–100%. Explants that disinfected the best were tubers and flower receptacles followed by leaf fragments and stolons (Table 4).

**Fig. 2:**
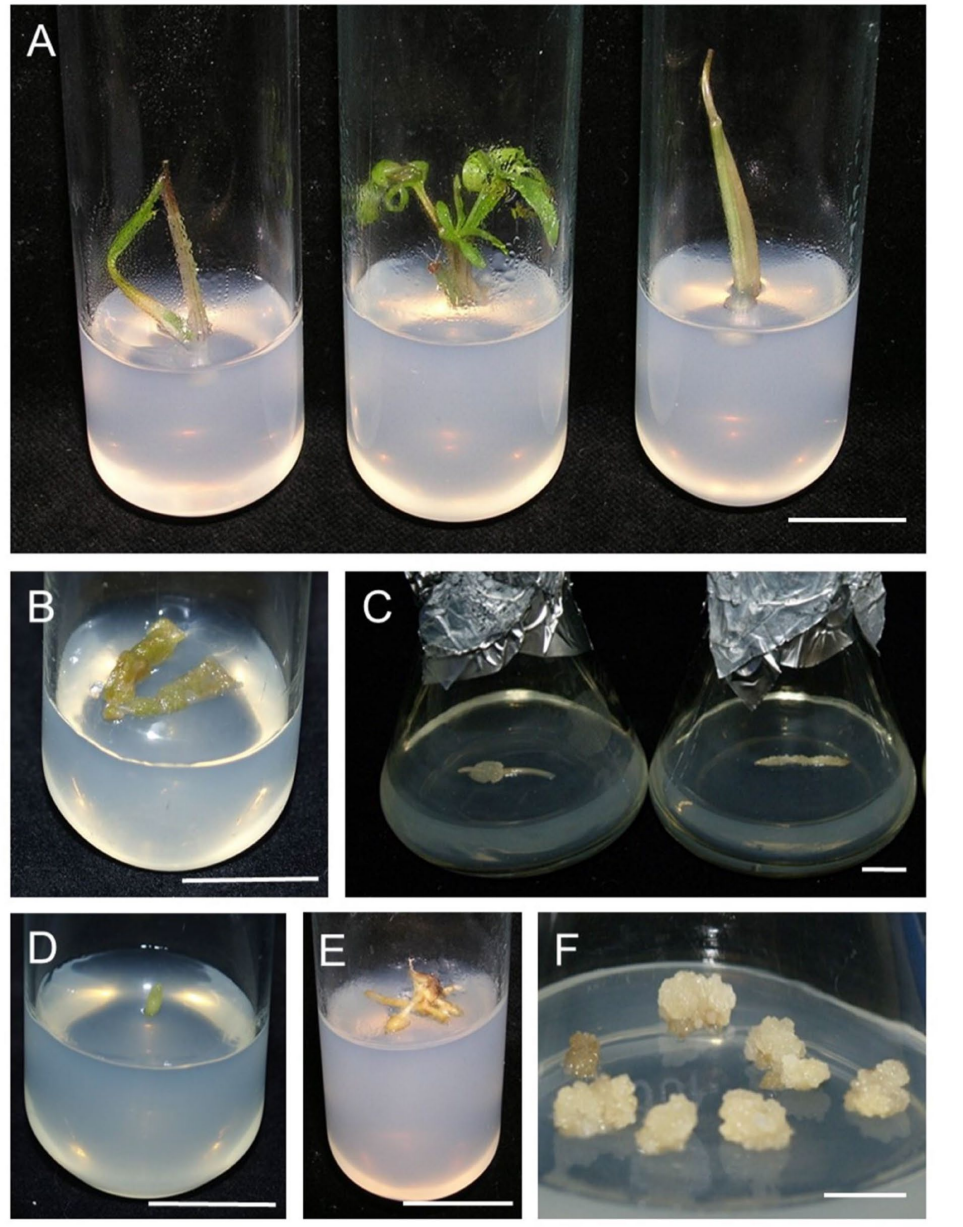
Development of various explants of *R. illyricus* on B_5_ mineral medium with MS micronutrients and various PGR combinations after 6 weeks of cultivation in A) photoperiod or B-F) darkness. (**A**) Apical buds on medium supplemented with 3.2 mg L^− 1^ NAA and 5.0 mg L^− 1^ kinetin; callus on the surface of (**B**) leaf and (**C**) fragments of stolons; (**D**) receptacle on medium with 8.0 mg L^− 1^ picloram and 8.0 mg L^− 1^ kinetin; (**E**) developing tubers on fragments of stolons on medium with 4.0 mg L^− 1^ 2,4-D and 8.0 mg L^− 1^ kinetin; (**F**) callus of stolon-origin on medium with 8.0 mg L^− 1^ picloram and 5.0 mg L^− 1^ kinetin, bar = 1 cm

Apical buds resumed growth regardless of what medium they had been inoculated on (Fig. 2A). Callus formation was observed on the other types of explants (Table 4). The earliest microcallus was noted on the surface of leaf explants (after 2–3 weeks; Fig. 2B). It was observed on leaves petioles after 3–4 weeks and the last it appeared on fragments of stolons – in the 6th week (Fig. 2C). The resulting callus was compact and light cream in colour and showed slow growth in the initial two subcultures.

**Table 4:** Results of the decontamination and growth reaction of different kinds of *R. illyricus* explants on media with different compositions of plant growth regulators

Type of explant	Auxins (mg L^− 1^)	Cytokinins (mg L^− 1^)	% of explants disinfected per isolated explants	% of callusing or growing* explants
Apical buds	NAA 3.2	Kinetin 5.0	100**	100
	NAA 2.5	BAP 2.5	75**	100
	Percentage of disinfection		87.5%	
Leaf	Picloram 8.0	Kinetin 3.0	50	0
	Picloram 8.0	Kinetin 5.0	82	50
	Picloram 8.0	Kinetin 8.0	90	80
	NAA 5.0	BAP 5.0	100	28
	NAA 2.5	BAP 2.5	100	0
	Percentage of disinfection		84%	
Petioles	Picloram 8.0	Kinetin 5.0	50	50
	Picloram 8.0	Kinetin 8.0	100	100
	NAA 5.0	BAP 5.0	83	33
	NAA 2.5	BAP 2.5	67	50
	Percentage of disinfection		75%	
Stolons	Picloram 8.0	-	80	80
	Picloram 8.0	Kinetin 3.0	85	54
	Picloram 8.0	Kinetin 5.0	86	86
	Picloram 8.0	Kinetin 8.0	71	71
	2,4-D 4.0	Kinetin 8.0	73	36
	NAA 5.0	BAP 5.0	100	0
	NAA 2.5	BAP 2.5	100	0
	Percentage of disinfection		85%	
Flower receptacle	Picloram 8.0	Kinetin 5.0	100	0
	Picloram 5.0	Kinetin 8.0	100	0
	Percentage of disinfection		100%	
Tubers	2,4-D 4.0	Kinetin 8.0	100	0
	Percentage of disinfection		100%	

*Growth refers to the apical buds, ** internal bacteria that gradually became visible in the following 2–3 subcultures

Isolated flower receptacles swelled, but no callus formation was observed (Fig. 2D). Tuber-like thickening developed on several explants taken from stolons (Fig. 2E), but no further growth or development occurred. No signs of dedifferentiation were observed on tubers, despite the absence of signs of decontamination damage.

### Callus cultures and indirect organogenesis

#### Experiment 1 - callus growth

A lower dose of kinetin stimulated a faster growth of the callus fresh matter (CFM) – the average increase was more than twice (Fig. 2F). The cytokinin concentrations used had no effect on callus dry matter (CDM) – Table 5.

**Table 5:** Callus fresh matter (CFM) and callus dry matter (CDM) gain on media with different concentrations of kinetin

Growth regulators (mg L^− 1^)		CFM%	CDM%
Picloram	Kinetin		
8.0	5.0	221.5 b*	9.4 a
8.0	8.0	148.4 a	10.8 a

*values within the column followed by the same letter are not significantly different at *p* = 0.05

#### Experiment 2 - callus growth and organogenesis

**Fig. 3:**
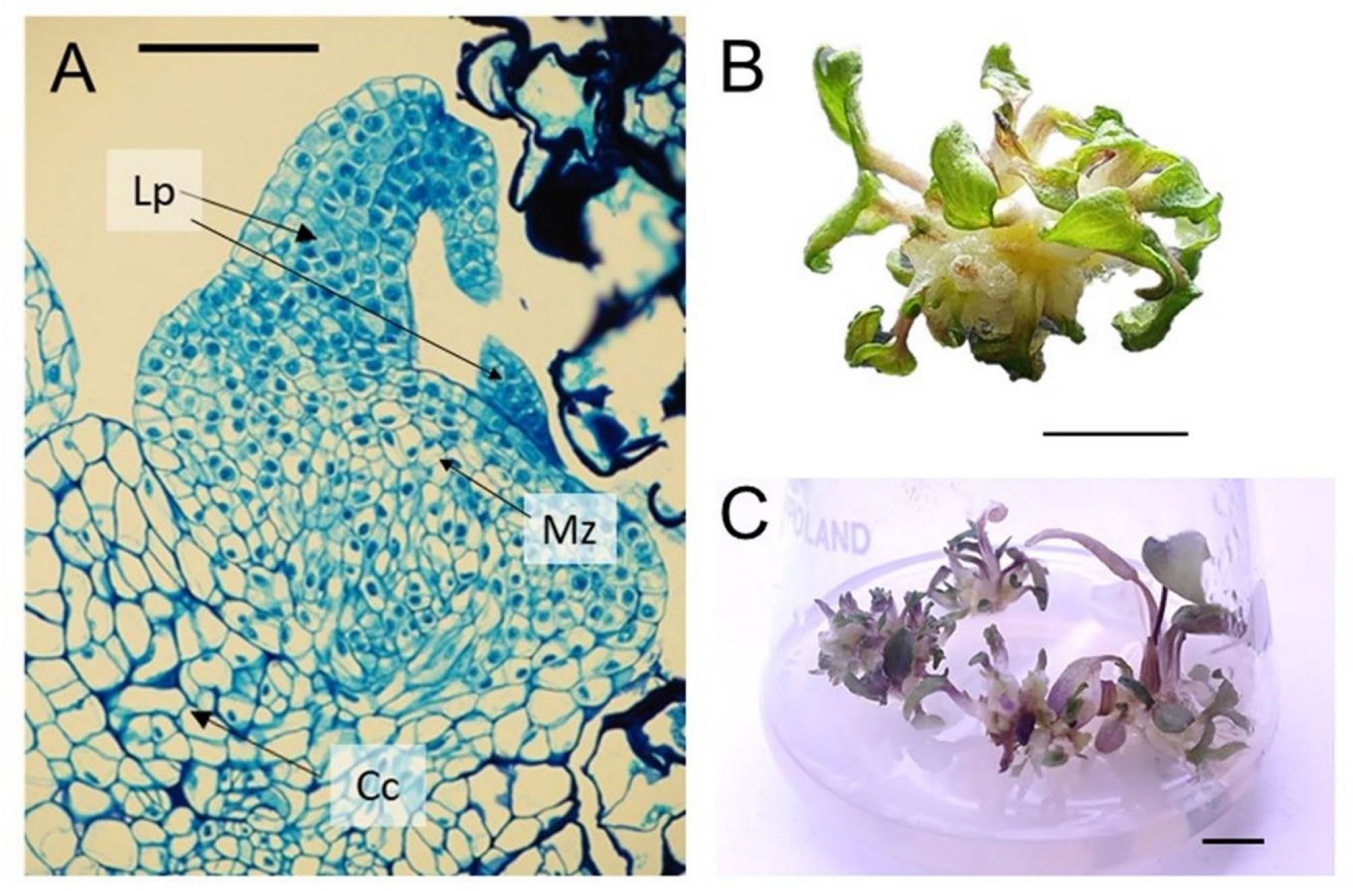
Regeneration of *Ranunculus illyricus* shoots in vitro. (**A**) section through stolon-origin callus with a differentiating adventitious bud after six weeks of cultivation on medium with 2.0 mg L^− 1^ IBA and 4.0 mg L^− 1^ BAP in darkness, Lp – leaf primordia, Cc - callus cell, Mz – meristematic zone, bar = 100 μm; (**B**) proliferating shoot bud after six weeks of cultivation on medium with 1.0 mg L^− 1^ IBA and 2.0 mg L^− 1^ BAP in photoperiod, bar = 1 cm; (**C**) cluster of adventitous buds/shoots with roots on the medium with 1.0 mg L^− 1^ IBA and 2.0 mg L^− 1^ BAP in photoperiod, bar = 1 cm 4.3. Multiplication and rooting of the shoots

The greatest callus growth was obtained on medium with the addition of 2,4-D auxin alone or picloram 6.0 mg L^− 1^ with kinetin 8.0 mg L^− 1^. The change in the ratio between auxin (picloram) and cytokinin (kinetin) in favour of the latter allowed identifying a medium that supported good callus growth in subsequent subcultures. However, differentiation was observed only on the medium with an addition of 2.0 mg L^− 1^ IBA and 4.0 mg L^− 1^ BAP. Anatomical cross sections (Fig. 3A) confirmed that there were adventitious buds, 6–10 per one gramme of callus (Table 6). They did not develop into shoots on the same medium. The medium without plant growth regulators did not support callus growth nor induce differentiation processes.

**Table 6:** Growth and organogenesis of *R. illyricus* on media with different combinations of plant growth regulators

Growth regulators (mg L^− 1^)		CMF%	Regeneration (number of shoots)
Auxins	Cytokinins		
-	-	57.3 a*	-
Picloram 4.0	Kinetin 8.0	127.8 a	-
Picloram 6.0	Kinetin 8.0	262.8 b	-
IBA 2.0	BAP 4.0	105.5 a	+ (6–10 shoots per 1 g of callus)
IBA 1.0	BAP 4.0	93.5 a	-
2,4-D 2.0	-	363.1 b	-

*explanation, see: Table 5

The isolated adventitious buds transferred to medium with a reduced level of growth regulators (1.0 mg L^− 1^ IBA and 2.0 mg L^− 1^ BAP) began to increase in size, producing new adventitious shoots and roots. From a single adventitious bud after six weeks of culture, 3–12 (average 7) shoots were formed (Fig. 3B). These shoots simultaneously rooted, producing 1–3 short roots (up to 2 cm). Having been transferred to fresh medium with a similar composition, both callus and adventitious buds retained their differentiation competence for more than two years.

### Hardening and acclimatisation of regenerates

**Fig. 4:**
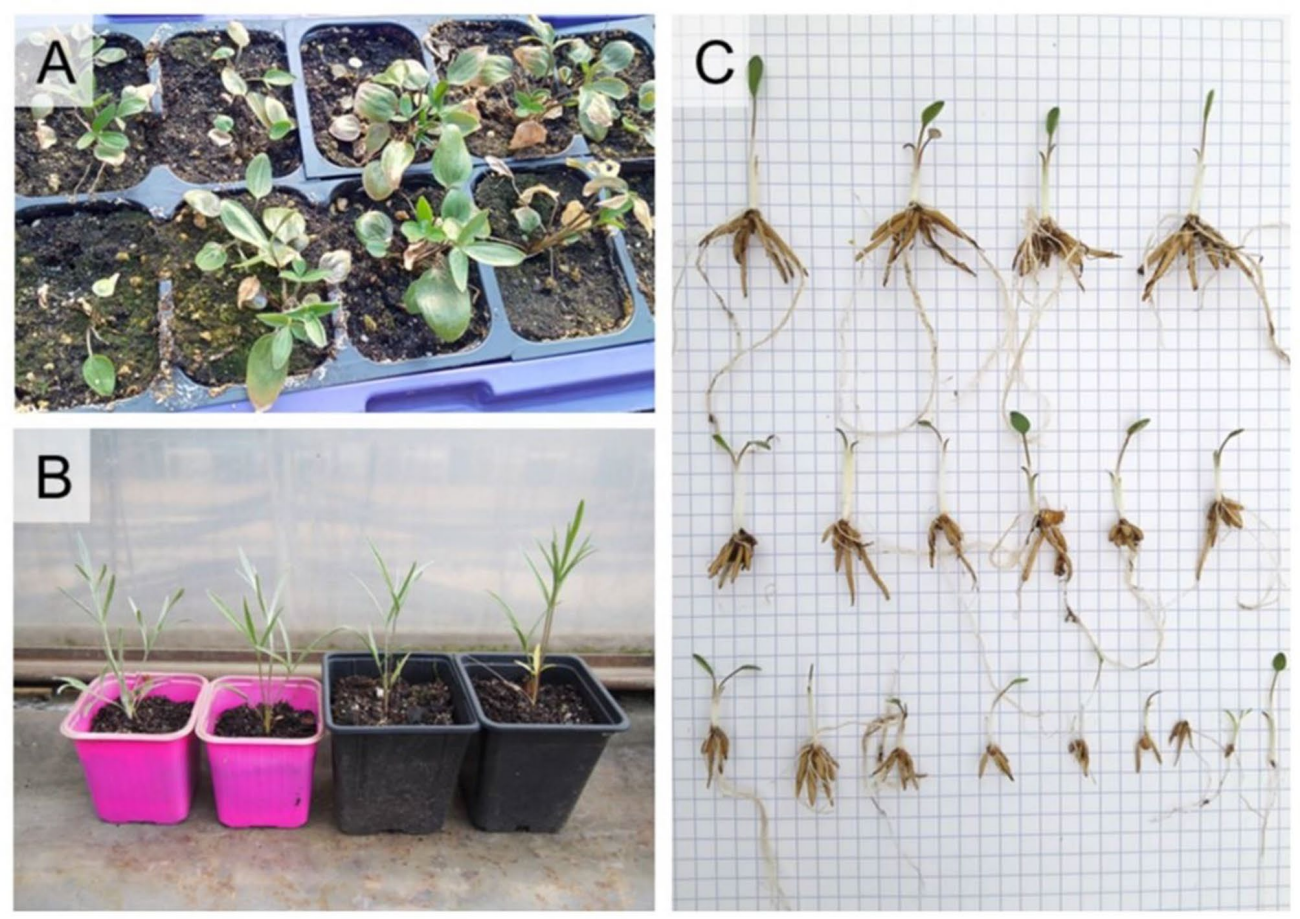
Acclimatisation of *Ranunculus illyricus* shoots. (**A**) Shoots after 8 weeks in Sanyo vegetative chambers; (**B**) Plants in the third (2023) growing season, left – in vitro origin, right – seed origin; (**C**) clusters of tubers developing at the base of in vitro origin shoots at the end of the first growing season

As many as 42% out of 74 hardened plantlets survived 8-week acclimatization process, and by autumn 2020 they had developed an average of 6 tubers in the cluster at the base of the shoot (dormant form of the plant, Kocot et al. 2022) – Fig. 4A, B, C. Of the planted tuber clusters, more than half (58%) survived the first winter and began to grow in spring 2021. At the end of the second growing season (2021), the average number of tubers in the cluster had reached 10 and 11% of the plants had developed clusters of daughter tubers at the ends of the growing stolons. The first flowering plants were observed in the third growing season.

### Ploidy assessment

**Fig. 5:**
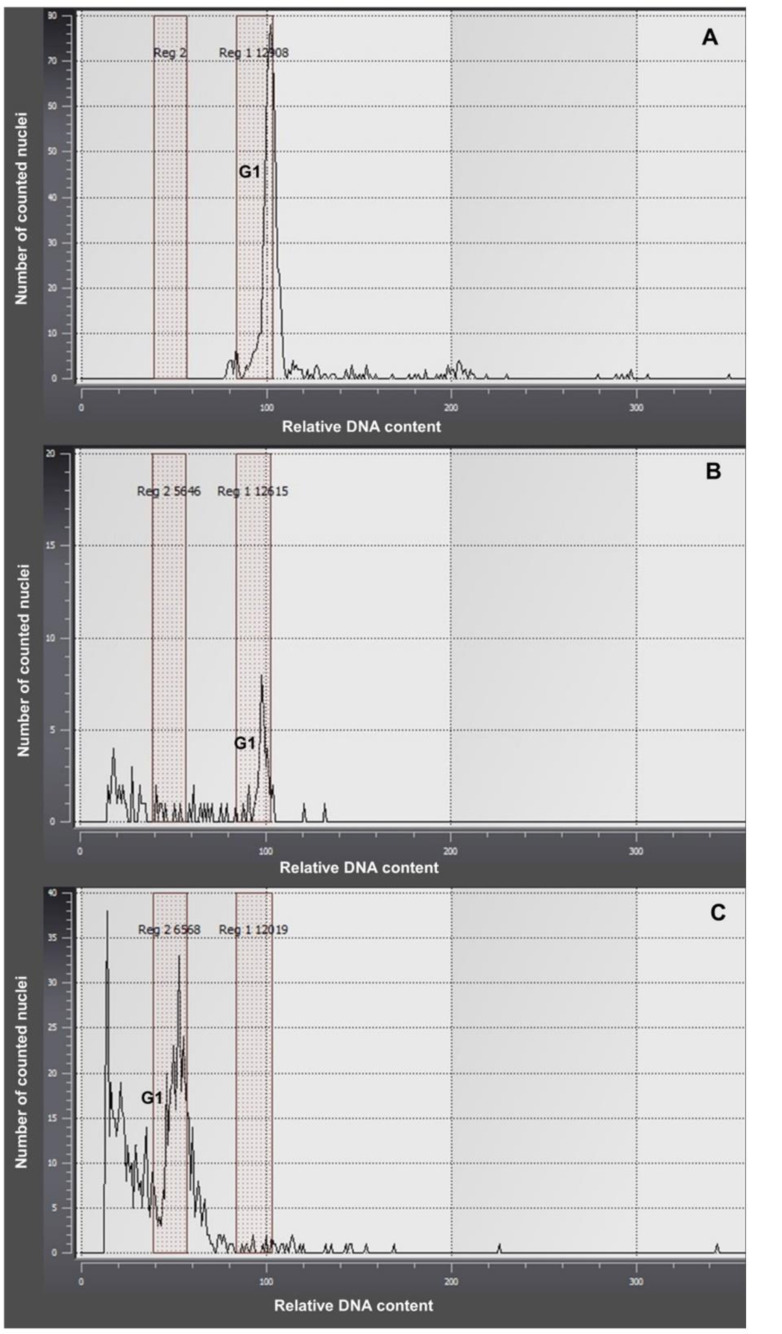
Histograms of relative DNA content in the nuclei of *Ranunculus illyricus* leaf cells: (**A**) control seed-origin plant; (**B**) tetraploid plant originating in vitro (indirect organogenesis); (**C**) diploid plant originating in vitro; G1, G2 — phases of cell life

The results of the flow cytometric analysis presented on the DNA histograms (Fig. 5) show a distribution of the relative DNA content with dominant peaks corresponding to the 2 C level in the G1 phase of the cell cycle of the seed-origin plant of *R. illyricus* (control plant, Fig. 5A). The DNA content analysed for nine out of ten plants after indirect organogenesis indicated that they did not differ from the seed-origin plants and that they were tetraploids (Fig. 5B). One regenerated plant appeared to be diploid (Fig. 5C).

## Discussion

The Illyrian buttercup, with its relatively large, intensely yellow flowers and silvery hairy leaves, is a species with great decorative potential. In addition, the short growing season, which allows avoiding the driest months, will make it possible to use this xerothermic species as ephemera in places with periodic water shortages. Unfortunately, this species is rare in many countries within its geographical range. In addition, it sets a small number of seeds, which despite their high vitality, germinate with difficulty [33]. For these reasons, obtaining plant material from nature for commercial propagation is very limited, and *ex vitro* generative reproduction is inefficient. Therefore, there is a need to elaborate propagation methods using in vitro techniques, both to meet commercial demands and as a resource of new genotypes, which can be selected or combined to obtain superior forms for cultivation. The first results that describe the in vitro propagation of *R. illyricus* are presented here.

For many reasons, seeds are the best starting material for establishing tissue cultures. However, in the case of the Illyrian buttercup, it was not possible to introduce plants into in vitro conditions in this way. The two most common factors that interrupt dormancy, the use of gibberellin [41] and the low germination temperature [42, 43], did not allow seedlings to be obtained despite the prolonged observation period of decontaminated achenes on the medium. The seeds were undoubtedly 100% viable (for details of tetrazolium test results see: [33]), so the negative impact of the disinfection process on their germination ability could be considered. Another reason could be the erratic reaction of seeds to previously effective dormancy breaking agents [33], which means that efforts to find a way to stimulate germination for this species should be continued. The initiation of *R. layalii* or *R. kazusensis* culture [19, 21] relied on the isolation of zygotic embryos after the removal of the pericarp and endosperm. However, when evaluating the viability of *R. illyricus* seeds following the terazolium method [33], the achenes were also deprived of the pericarp, and observations with the help of a binocular did not allow the embryo to be localised within the endosperm area (data not presented).

Another good way to initiate shoot cultures involves apical or axial buds, which easily pick up growth and proved to be efficient for *R. asiaticus* and *R. wallichianus* [17, 31]. However, permanently decontaminated buds for *R. illyricus* could not be obtained. Additionally, since Illyrian buttercup is a monopodial plant, the excision of such explants was tantamount to the plant destruction, and finally this strategy of culture induction was abandoned.

It is known that for some species of buttercups like *R. japonicus* [44] and *R. asiaticus* [26, 45, 46] it is possible to initiate cultivation from flower fragments. In our experiment, an isolated flower receptacle from immature *R. illyricus* flower buds failed to grow on the medium used (picloram + kinetin). On the other hand, a good source of Illyrian buttercup explants turned out to be the vegetative parts of plants, as previously proved for *R. scleratus* [15], *R. wallichianus* [16], *R. serbicus* [18], *R. casusensis* [20]. The decontamination process with varying effectiveness allowed aseptic explants to be obtained from above-ground parts (leaves, petioles) and underground parts (tubers, stolons). With the exception of tubers, callus appeared on all types of vegetative explants.

Contrary to expectations, the underground parts were easily decontaminated. Therefore, the callus of stolon-origin was selected for further differentiation experiments. The time when the above-ground parts are available for establishing cultures is relatively short; the plant blooms already in May and dies back. Stolons show a longer growth activity because they start to develop from a cluster of tubers in autumn and from that moment until May they can be a source of explants.

In previous works on in vitro regeneration of *Ranunculus* 2,4-D [31] or NAA [15, 20, 25, 31] were used. In some cases, the media were supplemented with cytokinins alone [17, 21, 31]. The most widely used cytokinin was kinetin combined with auxin but also with other cytokinins or as the only plant growth regulator. In the study presented here, picloram and kinetin were used at relatively high doses and for the dedifferentiation process of *R. illyricus* explants, the reduction in the cytokinin content was associated with a lower percentage of regenerating explants, both from leaf fragments and petioles (Table 4).

The callus regeneration procedure described here is the first to have used picloram for in vitro cultivation of the genus *Ranunculus*. This synthetic auxin has already been used in the cultivation of other dicots, for example, *Eysenhardia polystacha* or *Azadirachta indica* [47, 48]. Much more often it has been used for monocots cultivation, for example *Costus speciosus* [49], sometimes in high doses: 5 mg L^− 1^ in combination with kinetine was beneficial for the regeneration of kodo millet [50] or 10 mg L^− 1^ for seed germination and the regeneration of sugar cane callus [51].

The use of picloram at a dose of 8 mg L^− 1^ with 8 mg L^− 1^ of kinetin was more effective than kinetin at a dose of 5 mg L^− 1^ in the induction phase of the *R. illyricus* callus (Table 4), but not for further callus growth. The increase in fresh callus matter was greater on the medium with a lower dose of kinetin – 5 mg L^− 1^ (Table 5) and the change in proportion in favour of cytokinin (picloram 6 mg L^− 1^ and kinetin 8 mg L^− 1^) turned out to be the best for obtaininga large and sustained gain in callus CFM – Table 6. Such an intensely growing callus was homogenous, compact, but without signs of differentiation of adventitious buds. Another pair of regulators (IBA combined with BAP) induced organogenesis in the moderately growing callus (Table 6). *R. illyricus* required even lower concentrations of PGR (1.0 mg L^− 1^ IBA and 2.0 mg L^− 1^ BAP) for the buds to develop into shoots and roots on the same medium.

Spontaneous shoot rooting even without the use of PGR has been described for *R. layalii* [21] and *R. scleratus* [15]. The shoots of *R. asiaticus* and *R. wallichianus* [16, 31] had to be transferred to a medium with IBA auxin alone for rooting. The advantage of the procedure described for *R. illyricus* is that adventitious buds developed into shoots and rooted on the same medium.

It would seem that the efficiency of the acclimatisation process was not high (42%) and only more than half of the plants survived the first winter in open air conditions. However, the efficiency of acclimatisation can be compared with the process of obtaining *R. illyricus* plants from seeds: the highest seed germination rate of this species was 40%, and the percentage of seedlings surviving the first winter was 50% [33]. Therefore, the efficiency of obtaining new individuals in these two cases – plants of generative origin and after in vitro propagation – is comparable. Micropropagated plants already in the first season of *ex vitro* vegetation began to produce tuber clusters at the base of the shoot (Fig. 3C), while seedlings only in the second season. Both seedlings and plants of in vitro origin bloom for the first time in the third growing season. It turns out that in vitro cloning could be at least as effective as *ex vitro* generative reproduction. Furthermore, taking into account the potential number of microplants developed during consecutive subcultures, the in vitro method is even more effective and the resultant plants are of similar quality (Fig. 3B).

Ploidy assessment of *R. illyricus* showed that in vitro cultivation favours the appearance of somaclonal variation. Somaclonal variation, badly perceived in the context of species conservation, offers great breeding potential, especially in the case of species where generative reproduction is not very effective, such as the Illyrian buttercup. Somaclonal variants were already used in the breeding of another *Ranunculus* species of similar reproductive biology – *Ranunculus asiaticus* [46] or *R. lyallii* [21]. It seems that an accurate evaluation of the true quality is necessary for each micropropagated plant of *R. illyricus* so that selected individuals can be used properly: for breeding or for horticultural and conservation action.

The species used in urban greenery should be decorative, but also adapted to the conditions they must meet in the intensifying phenomenon of the urban heat island. In this context, the Illyrian buttercup is a valuable plant. Due to its structure and biology, the plant is suitable for the urban environment because of both its adaptations and decorative qualities. Moreover, urban habitats are suitable for the Illyrian buttercup – an endangered species - as places of conservation.

City parks, woods, lawns, wetlands, and riverbanks can provide microhabitats for various rare and endangered species. High environmental heterogeneity in urban areas can be helpful in

*ex situ* conservation program and complement* in situ* conservation carried out in nature reserves. There are examples of plants threatened with extinction introduced into the urban environment, for examples, *Mosla hangchowensis* [52] or *Calycanthus chinensis* [53]. Another interesting example is the population of the rare plant *Sternbergia colchicifora* found along a busy road section in the downtown of the county seat Veszprém (Hungary). Within this anthropogenic habitat of traffic islands, in the vicinity of roads and sidewalks where *S. colchicifora* occurs, other rare plants (e.g., *R. illyricus*) [54] have been found. It shows that urban green sites could be a valuable way of *ex situ* protection in the case of this species. Illyrian buttercup has also been discovered in other synanthropic habitats, such as burial mounds [55] or old graveyards [56]. The plant material obtained through indirect organogenesis as a result of our research could be used to create *ex situ* conservation sites in urban areas.

The idea of using native wild species in urban greenery has become more and more popular in recent times. In this way, the introduction of alien and potentially invasive species is avoided. It is worth noting that native wild species are also a valuable source of food for pollinators in urban areas. Ranunculaceae species are recommended for cultivation in urban space as they provide a continuous flowering sequence and are a pollen source, especially in early spring [57]. They are eagerly visited by various species of insects, so they should also be taken into account when planning the protection of pollinating insects [58]. In the urban area of Berlin, 179 endangered plant species were listed, of which 10 belonged to the Ranunculaceae family [59].

## Conclusions

This paper introduces for the first time the effective method of in vitro propagation of *R. illyricus* – a xerothermic species endangered in some European countries and with high ornamental potential in urban space. The complete protocol for long term in vitro cultivation of *R. illyricus* was elaborated. The best starting explants for establishing an in vitro culture turned out to be fragments of underground stolons taken in spring. The excision of such explants does not damage the mother plant and such explants were easily disinfected using 0,1% mercuric chloride for 5 min. Although they were the last among different types of explants to start the dedifferentiation process, the callus obtained from them grew the best. The best medium for stolon dedifferentiation was the medium containing macroelements of B_5_ with microelements MS supplemented with 8.0 mg L^− 1^ of picloram and 5 mg L^− 1^ of kinetin while the cultivation took place in the dark. The increase in fresh matter of cultivated callus was the highest on the medium with the addition of 2.0 mg L^− 1^ of 2,4 – D in subsequent passages and was up to 363.1%. The first adventitious buds in the callus differentiated on medium with 2.0 mg L^− 1^ IBA and 4.0 mg L^− 1^ BAP. After transferring to the medium with the PGR dose reduced by half − 1.0 mg L^− 1^ IBA and 2.0 mg L^− 1^ BAP - a culture of buds and adventitious shoots was obtained, which simultaneously multiplied and rooted. The acclimatized shoots were planted into a mixture of perlite and commercially available potting soil, and the acclimatized shoots were initially kept for 2 months in a chamber with gradually decreasing humidity from 70% and then moved to the open air condition. A notable advantage of this method is the growth, multiplication, and rooting of adventitious shoots on the same medium. The quality of the plants that originated from in vitro and the acclimatisation efficiency are comparable to those seen in seedlings production. The plants developed well when grown in urban conditions, and the created population was constantly expanding, mainly through vegetative reproduction. However, if the number of in vitro multiplication passages per year and the low efficiency of seed setting are taken into account, in vitro cultures are a more effective method of *R. illyricus* reproduction. Obtaining plants using the described micropropagation method may becomea valuable complement to the *ex situ* conservation program of a species, that can be propagated both vegetatively and generatively, providing that dormancy problem of viable seed will be handled. Observations of cultivation in urban conditions and literature reports indicate the possibility of establishing replacement sites in urban areas for *ex situ* protection. The plant material described herein could serve as an attractive addition to urban greenery, enriching the supply of decorative plants resilient to challenging urban conditions, and could be used in breeding.

## Data Availability

The datasets used and/or analysed during the current study are available from the corresponding author on reasonable request.

## Abbreviations

2,4 –D: 2,4-Dichlorophenoxyacetic acid )
B_5_: Gamborg mineral medium
BAP: 6-Benzylaminopurine
CDM: callus dry matter
CFM: callus fresh matter
FMf: final fresh matter of callus (mg)
FMi: initial fresh matter of callus (mg)
GA3: Gibberellic acid
IBA: Indole-3-butyric acid
MS: Murashige and Skoog mineral medium
NAA: 1-Naphthaleneacetic acid
